# Homeostatic status of thyroid hormones and brain water movement as determinant factors in biology of cerebral gliomas: a pilot study using a bioinformatics approach

**DOI:** 10.3389/fnins.2024.1349421

**Published:** 2024-02-27

**Authors:** Carmelita Bastos Mendes, Lanni Sarmento da Rocha, Carlos Alberto de Carvalho Fraga, Adriana Ximenes-da-Silva

**Affiliations:** ^1^Laboratório de Eletrofisiologia e Metabolismo Cerebral, Instituto de Ciências Biológicas e da Saúde, Universidade Federal de Alagoas, Maceió, Brazil; ^2^Faculdade de Medicina, Universidade Federal de Alagoas, Arapiraca, Brazil

**Keywords:** gliomas, AQPs, water movement, biomarkers, gene expression, thyroid hormones, correlation analysis

## Abstract

**Introduction:**

The expression and localization of the water channel transporters, aquaporins (AQPs), in the brain are substantially modified in gliomas during tumorigenesis, cell migration, edema formation, and resolution. We hypothesized that the molecular changes associated with AQP1 and AQP4 in the brain may potentially be anticancer therapeutic targets. To test this hypothesis, a bioinformatics analysis of publicly available data from international consortia was performed.

**Methods:**

We used RNA-seq as an experimental strategy and identified the number of differential *AQP1* and *AQP4* transcript expressions in glioma tissue compared to normal brain tissue.

**Results:**

AQPs genes are overexpressed in patients with glioma. Among the glioma subtypes, AQP1 and AQP4 were overexpressed in astrocytoma (low-grade glioma) and classical (high-grade glioma). Overall survival analysis demonstrated that both AQP genes can be used as prognostic factors for patients with low-grade glioma. Additionally, we observed a correlation between the expression of genes involved in the tyrosine and thyroid hormone pathways and AQPs, namely: *PNMT, ALDH1A3, AOC2, HGDATP1B1, ADCY5, PLCB4, ITPR1, ATP1A3, LRP2, HDAC1, MED24, MTOR*, and *ACTB1* (Spearman’s coefficient = *geq* 0.20 and *p*-value = ≤ 0.05).

**Conclusion:**

Our findings indicate that the thyroid hormone pathways and AQPs 1 and 4 are potential targets for new anti-tumor drugs and therapeutic biomarkers for malignant gliomas.

## 1 Introduction

Gliomas are the most common central nervous system tumor type accounting for approximately 80% of all malignant intracranial tumors with high episodes of recurrence (Ostrom et al., 2014).

Depending on the cell type of origin, they can be classified or subdivided into astrocytomas, ependymomas, oligodendrogliomas, oligoastrocytomas, and glioblastoma multiforme (GBM) (Ostrom et al., 2014).

*Glioblastoma* is the most aggressive brain tumor owing to its high invasiveness. Despite advances in therapeutic modalities (tumor resection combined with chemotherapy and radiotherapy), the overall patient survival rate is 12–15 months from the initial diagnosis, with only 5% of patients reaching a 5-year survival rate (Fedele et al., 2019).

Likewise, temozolomide (TMZ), a DNA alkylating agent, is the standard first-line treatment for GBM, has a significant tumor recurrence rate and confers drug resistance (Strobel et al., 2019) due to its low specificity and limitations in crossing the blood-brain barrier (BBB).

Persistent headaches, neurological and behavioral deficits, seizures, cognitive deficits, drowsiness, dysphagia, confusion, aphasia, motor deficits, fatigue, dyspnea, and mood changes are common symptoms in patients diagnosed with glioblastoma (Maugeri et al., 2016; IJzerman-Korevaar et al., 2018). Usually, its localization, size, and grade make it inoperable and difficult to treat, drastically decreasing the quality of life of patients with glioma (Fedele et al., 2019).

Many symptoms are derived from neurological findings such as increased intracranial pressure and brain herniation. Edema around the tumor causes hypoxia (lack of oxygen supply) (Solar et al., 2022), resulting in long-term disability, psychiatric disorders, substance abuse, or self-harm (Huang et al., 2021).

A clinical study carried out by Wu et al. (2015) showed that within the identification of peritumoral edema during the preoperative period in patients with glioblastoma, factors such as necrosis and edema extent were independent factors for poor outcome and overall survival (OS).

Aquaporins (AQPs), a family of transmembrane water channel proteins, play a critical role in responding to changes in the osmotic environment. Eight members of the water channel family are expressed in the central nervous system (Saadoun et al., 2002; Maugeri et al., 2016). AQP1 and AQP4 are the major water channels in the brain and play a pivotal role in water homeostasis and the maintenance of BBB integrity (Galán-Cobo et al., 2016).

Brain AQPs are distributed primarily in astroglial membranes, especially in perivascular astrocyte foot processes and glia limitans, near the brain fluid compartments (Hubbard et al., 2018; Li et al., 2019) and ependymal cells, providing an efficient system to promote water and solute transport between the perivascular space and glial cells, and to support brain parenchymal clearance through the glymphatic system (Smith et al., 2017; Mestre et al., 2018; Lohela et al., 2022).

AQP4, an orthodox AQP type, is upregulated in gliomas and is involved in the tumorigenesis process, that is, cell migration, invasion, and functional changes in the surrounding tissue. AQP4 distribution has been hypothesized to underlie mechanisms related to the degree of malignancy (Lan et al., 2017), edema formation in the peritumoral region, increased intracranial pressure, and seizure episodes (Dubois et al., 2014; Maugeri et al., 2016).

Tumor cell migration is a complex mechanism not well understood. However, angiogenesis is a critical feature differentiating high-grade and low-grade glioma. Glioblastoma as the most aggressive brain tumor shows morphological and vascular changes characterized by a high invasiveness and migration, which are associated with the expression of AQP1 and AQP4 and mesenchymal transformation as previously described (Hardee and Zagzag, 2012; Brennan et al., 2013).

Aquaporins have been described to have a vital role in enhanced invasion and tumor cell migration as reviewed by De Ieso and Yool (2018) and Moon et al. (2022). Of special importance is the role of AQP1 and -4 in the proposed mechanism of invasiveness and migration of glioma cells. During tumor cells metastasis modifications of blood supply, cytoskeleton and extracellular matrix structure are needed to promote angiogenesis for tissue demands of oxygen and nutrient delivery. AQP1 was found to boost endothelial cell migration and, in association with overexpression of AQP4 to regulate water influx and extracellular matrix interaction leading to tumor cell membrane filopodia formation, mechanisms linked to metastasis progression.

Several *in vitro* and *in vivo* studies have reported reduced invasive capacity and migration in tumors after AQP4 depletion or downregulation (Ding et al., 2010, 2011; Cheng et al., 2017) by decreasing water permeability, which in turn upregulates transmembrane water fluxes during tumor or healthy astroglial cell movement (Huang et al., 2021).

In contrast, AQP1 (also considered a classic aquaporin) is predominantly expressed in the circumventricular brain and areas associated with cerebrospinal fluid production. Its expression is altered in the human brain under pathological conditions (Lima et al., 2012) such as cancer. In brain tumors, AQP1 and AQP4 expression is upregulated with the malignancy grade (Saadoun et al., 2002).

Inhibition of tumor growth and suppression of vasculogenic mimicry formation were observed following *AQP1* silencing *in vivo*, which could be related to reduced tumor aggressiveness, malignancy, and metastasis (Perry and Wesseling, 2016).

More recently, the newly updated World Health Organization (WHO) Classification of Tumors of the Central Nervous System classifies brain tumors based on their histological appearance and molecular markers (genotypic classifications). Namely: isocitrate dehydrogenase (IDH) mutation, 1p/19q codeletion, and O-6-methylguanine-DNA methyltransferase (MGMT) methylation, avoiding imprecise diagnosis, thereby significantly influencing in the selection of treatment options for patients (Martinez-Lage and Sahm, 2018; Huang et al., 2020).

Owing to the role of molecular markers in the early diagnosis of gliomas, we hypothesized that AQP1 and AQP4, as homeostatic brain proteins, could act as potential anticancer therapeutic targets.

In this study, we aimed to characterize the expression patterns of genes encoding AQP1 and AQP4 proteins and to identify the genes present in the cancerous microenvironment that potentially regulate or correlate with their expression in human gliomas. Our findings, from a bioinformatic approach, allowed us to conduct a wide and diversified scale analysis and provided evidence for the role of these proteins and other related genes, thus paving the way for the development of potential biomarker candidates for the diagnosis and therapeutic targets of glioma for future research.

## 2 Materials and methods

### 2.1 Experimental design

This study was executed using publicly available data from international consortia. In this study, RNA-seq was used as the search strategy. Differential expression analysis was performed using Bioconductor statistical packages with R software (version 4.1). Genomic data on human cancer samples originated from The Cancer Genome Atlas (TCGA) and healthy tissue of the Genotype-Tissue Expression (GTEx) project.

We used web tools to assess the variables beyond differential expression. Correlation analysis was performed using Gene Expression Profiling Interactive Analysis (GEPIA2) (Tang et al., 2019),^1^ survival analysis, and Tumor IMmune Estimation Resource (TIMER) (Li et al., 2020).^2^ In addition, we used the Atlas of Human Pathology^3^ to visualize the distribution pattern of AQP1 and AQP4 proteins in healthy brain and cancer tissues.

The database annotation, visualization, and integrated discovery (DAVID) web server (Sherman et al., 2022)^4^ was used to identify a network of related genes in altered pathways in the tumor microenvironment (functional enrichment analysis). The same analysis was performed using PathfindR package (Ulgen et al., 2019).

In a short time, the genes involved in the pathways of our choice were evaluated by the degree of correlation with *AQP1* and *AQP4* expression in glioma tissues, both low-grade glioma (LGG) and GBM, to find similarities in expression profiles and suggest some degree of co-regulation.

Clinical data from patients regarding brain tumor location were not accessed and were not included in this study.

### 2.2 Transcriptional expression of *AQP1* and *AQP4*

Genetic profiles of normal and solid primary tumor tissues for the two types of cancer were downloaded from TCGA^5^ using TCGAbiolinks R package version 2.12.6.

A dataset composed of 2,080 RNA-seq of both tumor stages (LGG and GBM, *N* = 671) and healthy patient samples (*N* = 1,409) from TCGA and GTEx projects was used. Data were derived from the Illumina HiSeq RNA-Seq platform. The transcript levels of orthodox AQPs (*AQP1* and *AQP4*) in tumor and non-tumor tissues were evaluated. All data were processed and standardized using R software version 4.1.0.

The GEPIA2 web-based tool was used to build graphs to better visualize the results. One-way analysis of variance (ANOVA) was used for the assessment of the disease state: tumor, *N* = 518 (LGG) and *N* = 163 (GBM), or normal brain cortex, *N* = 207, as the variable for calculating the differential expression. The expression data were first transformed using the formula log2 (TPM + 1).

The fold change (log2FC) was defined as the difference in the median value between the normal and tumor samples. Genes with *P* ≤ 0.05 and | log2fold change (FC)| ≥ 1.0 were considered differentially expressed genes (DEGs). The same analysis was performed for glioma subtypes. All the results are presented in table and box plot forms.

The values were calculated using the corresponding formula:


l⁢o⁢g⁢2⁢F⁢C=l⁢o⁢g⁢2⁢(B)-l⁢o⁢g⁢2⁢(A)

Where B and A are representative of the median values of the genes expressed in the tumor and median values of the expressed genes in normal tissue, respectively.

### 2.3 Survival analysis

We sought to assess whether our search goal genes in this study may have survival advantages and, thus, establish possible prognostic markers, confirming their clinical significance.

Patients with glioma were divided into high- or low-level groups based on the median value of genes, and OS rates were evaluated using Kaplan–Meier analysis. The TIMER tool used the log-rank test (Mantel-Cox test) for the hypothesis evaluation. The Cox proportional hazard ratio (HR) and 95% confidence interval (CI) were used as parameters in the Kaplan–Meier survival analysis. Statistical significance was set at *P* < 0.05.

### 2.4 Functional enrichment analysis

Kyoto Encyclopedia of Genes and Genomes (KEGG) is an integrated database resource for biological interpretation of genome sequences and other high-throughput data. KEGG analyses are available in the DAVID database (see text footnote 4), a data resource composed of an integrated biology knowledge base and analysis tools to extract meaningful biological information from large quantities of gene and protein collections. Dysregulated pathway identification analysis was performed on differentially expressed genes using the DAVID web tool. The DEG analysis showed 4,820 genes in glioma samples (see Supplementary material) that were divided into two groups of lists: upregulated and downregulated genes (logFC > 1 and logFC < 0). Subsequently, these lists were submitted separately to DAVID and the results were generated.

### 2.5 Correlation analysis using the GEPIA2 tool

A pairwise gene Spearman correlation analysis between AQP*1 or AQP4* and the expression of other preselected genes was performed using GEPIA2.

GEPIA data are presented as a log-scale axis and a non-log scale was used for calculation; the strength of the correlation was determined using the following guide for the absolute value: ≤0.2 (weak), 0.21–0.50 (moderate), 0.51–0.80 (strong), and 0.81–1.0 (very strong). Spearman’s coefficients >0 indicate a positive correlation, and coefficients less than zero indicates a negative correlation. Spearman’s coefficient ≥0.20 and *p-*values ≤0.05 were considered significant in the current study.

## 3 Results

### 3.1 *AQP1* and *AQP4* genes are overexpressed in gliomas compared to normal brain samples

To characterize gene expression, differential expression analysis from TCGA and GTEx datasets was performed using the R software. This analysis revealed a list of 4,819 genes. Among these, five *AQPs* family members were identified: *AQP1*, *AQP4*, *AQP5*, *AQP7*, and *AQP8*.

**FIGURE 1:**
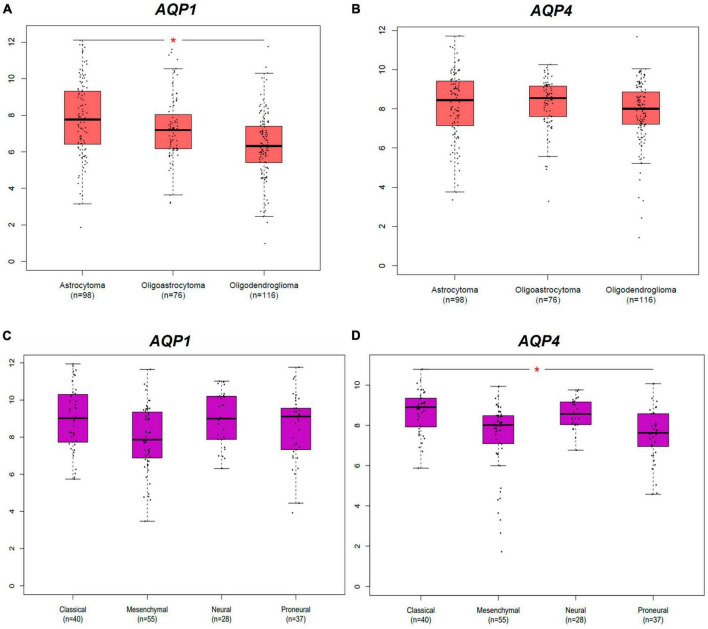
Differential expression AQPs mRNAs from gliomas samples from TCGA and GTEx dataset. AQP1 and AQP4 genes. Tumor samples are divided into LGG and GBM subtypes groups. **(A,B)** Represents both AQP1 and AQP4 profiles of expression in LGG subtypes. **(C,D)** Represents both AQP1 and AQP4 profiles of expression in GBM subtypes. LGG, low-grade glioma; GBM: glioblastoma multiforme. *p < 0.05. All graphs were derived by the GEPIA2 webserver in January 2021.

**FIGURE 2:**
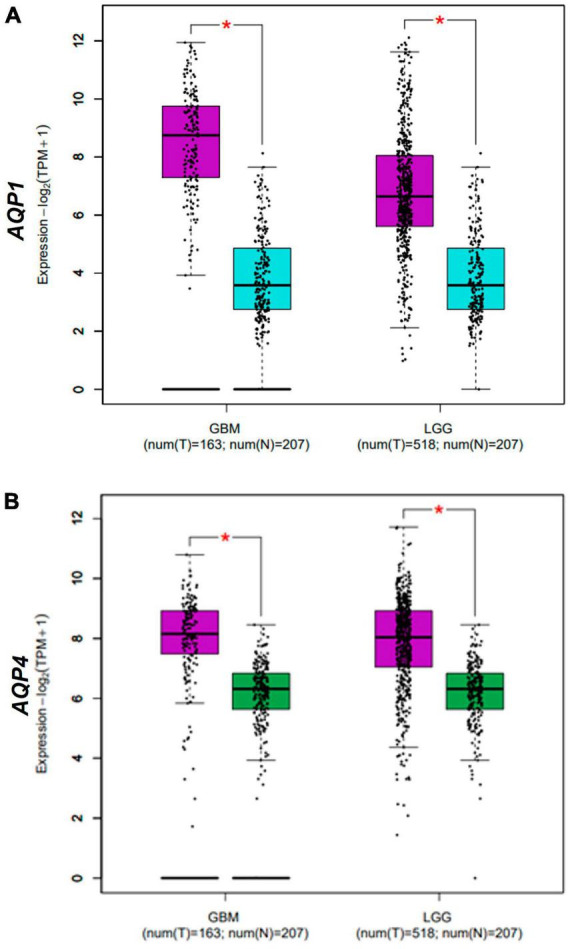
Differential expression AQPs mRNAs from gliomas samples from TCGA and GTEx dataset. *AQP1* **(A)** and *AQP4* **(B)** genes in normal (*N* = 163; blue box for AQP1 data and green box for AQP4 data) and tumor (*N* = 207; purple box) samples. Tumor samples are divided into LGG and GBM groups (*X*-axis of both graphs). TPM, transcripts per kilobase million; LGG, low-grade glioma; GBM, glioblastoma multiforme. *p < 0.05. Graphs **(A,B)** were derived by the GEPIA2 webserver in January 2021.

As expected, both *AQP1* and *AQP4* mRNAs were overexpressed in glioma samples (logFC = 1.42 x 10+14 and 1.02 x 10+14; *P*-value = 1.66E-40 and 8.82E-26, respectively) compared with the healthy brain samples (Table 1 and Figures 1A, B, 2).

**TABLE 1:** AQP’s differential expression in gliomas samples from TCGA and GTEx dataset.

GENE ID	LogFC	*P*-Value	Adjusted *P*-value
*AQP1	1.42	1.66E–40	3.15E–40
*AQP4	1.02	8.82E–26	1.48E–25
*AQP5	−2.18	1.15E–100	3.51E–100
*AQP7*	−3.11	1.1E–114	3.76E–114
*AQP8*	−2.19	7.71E–124	2.83E–123

*Orthodox aquaporins: functional division of AQPS. This group is permeable to water, but not to small neutral solutes. Other members of the family of these proteins are aquaglyceroporins (Yasui et al., 1999; Soveral et al., 2010).

**FIGURE 3:**
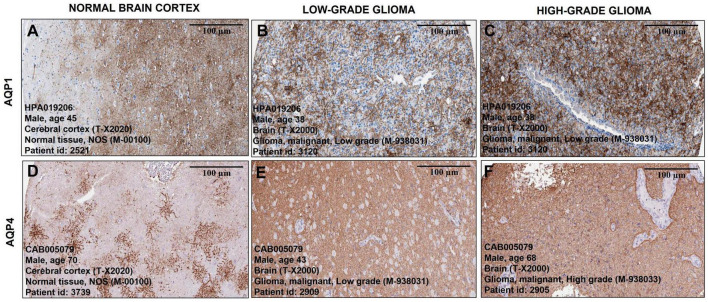
Representative immunohistochemical staining distribution of AQP1 and AQP4 expressions in normal and glioma patient samples. Plates **(A–F)** are captured in 100 μm. It is important to highlight that the staining (brownish) of the antibody for both proteins becomes more pronounced and denser as the degree of tumor malignancy increases compared to representative images of healthy brain tissue **(A,D)**. All data were extracted from the Human Protein Atlas (https://www.proteinatlas.org/).

Histological samples of tumor and normal brain tissues from the Human Protein Atlas database show the distribution of AQP1 and AQP4 proteins. The staining intensity of both proteins increased with the degree of tumor malignancy. Figures 3A–C show AQP1 staining, while Figures 3D–F show AQP4 staining.

**FIGURE 4:**
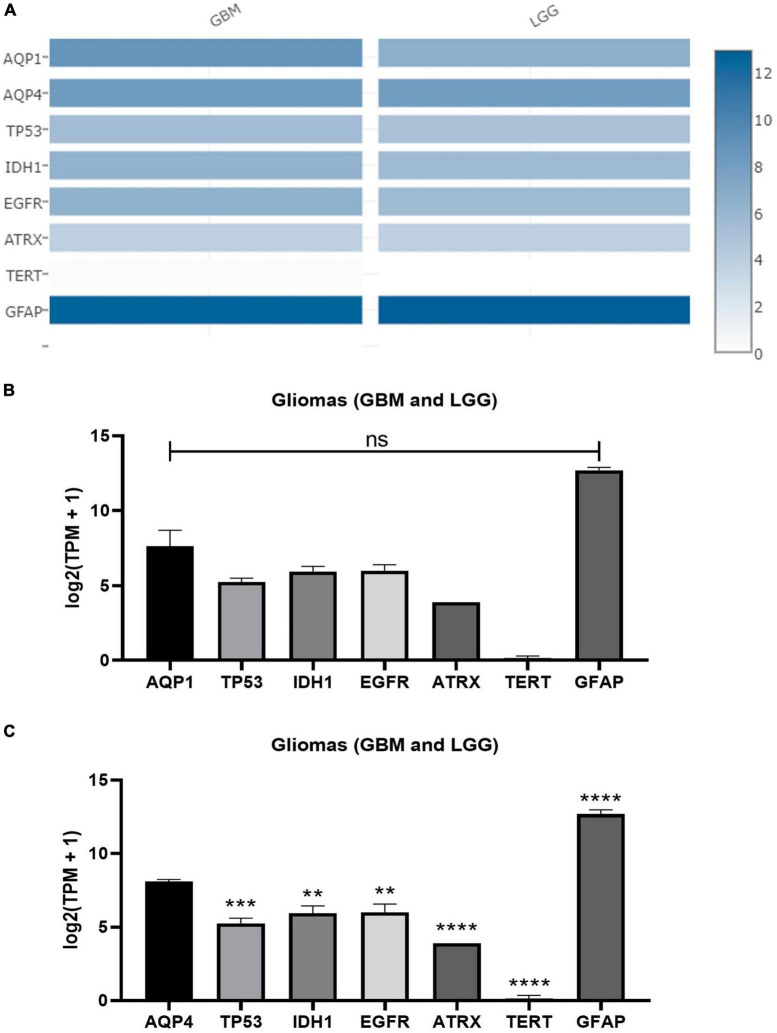
Expression matrix graphs based on a list of genes considered biomarkers of glioma and AQP1 and AQP4. **(A)** Heat Map derived from GEPIA2. The color density in each block represents the median value of gene expression in tumor tissue (GBM and LGG, separately) normalized by the maximum median value of the expression in all blocks. These data were transformed for plotting [linear to log2(TPM + 1)]. Graphs **(B,C)** were created from the values provided by the heap map for each gene with a subsequent comparison with both AQP1 and AQP4, respectively. For this, one-way ANOVA was performed followed by Tukey’s multiple comparisons tests using GraphPad Prism version 8.0. **p < 0.01, ***p < 0.001, ****p < 0.0001. TP53, tumor protein P53; IDH1, isocitrate dehydrogenase [NADP(+)] 1; EGFR, epidermal growth factor receptor; ATRX, ATRX chromatin remodeler; TERT, telomerase reverse transcriptase; TPM, transcripts per kilobase million; GFAP, glial fibrillary acidic protein.

In addition, both AQP1 and AQP4 showed a significant difference in expression pattern upon comparison with tumor markers/antigens in gliomas that are consensually used in clinical practice to establish degrees of malignancy and prognosis (Soldatelli et al., 2022). For instance: TP53, ATRX, TERT, IDH1, EGFR, and GFAP (Figure 4).

These results revealed a pivotal role of the above-mentioned genes in the gliomagenesis process.

### 3.2 *AQP1* and *AQP4* seem to play substantial roles in the molecular glioma subtype

In a complementary manner, Figure 2 shows the differential expression of AQP1 and AQP4 in glioma subtypes. A and B represent the expression patterns in the LGG subtypes (astrocytoma, oligoastrocytoma, and oligodendroglioma). In this case, AQP1 showed an increased number of transcripts in astrocytomas compared than in oligodendroglioma tumors (*p ≤ 0.05) (Figure 2A). AQP4 mRNAs levels were not significant in these LGG samples (Figure 2B).

Regarding the GBM subtypes (classical, mesenchymal, neural, and proneural), only AQP4 presented considerable variation in its expression between classical and proneural subtypes, being more expressed in the classical case (*p ≤ 0.05) (Figure 2D). AQP1 mRNAs levels were not significant in these GBM samples (Figure 2C).

These results indicate that the aquaporins in question most likely have an oncogenic role only in cells of astrocytic origin.

### 3.3 *AQP1* and *AQP4* could be potential risk factors and prognostic indicators for patients with low-grade glioma

**FIGURE 5:**
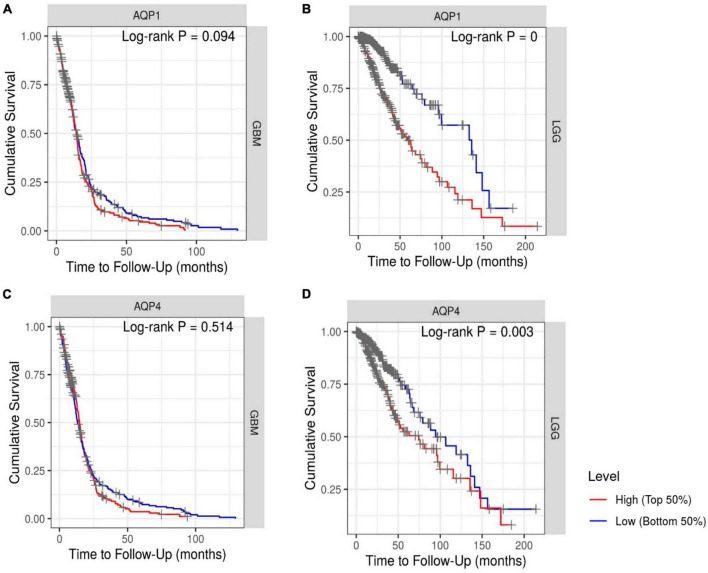
Kaplan–Meier survival analysis. **(A,C)** Patients with GBM and **(B,D)** patients with LGG. The Cox proportional hazard ratio (HR) and the 95% confidence interval were used in the Kaplan–Meier survival analysis. Red and blue curves represent high and low expression profiles, respectively. GBM, *N* = 523 patients with 448 dying. LGG, *N* = 514 patients with 125 dying. Log-rank *P*-values ≤0.05 were considered statistically significant. All graphs were derived by the TIMER database in September 2021.

One of the factors in assigning clinical importance to a therapeutic target is the probability of increasing the chances of patient survival. Kaplan–Meier analysis revealed that OS was lower in patients with LGG who had high *AQP1* (HR = 1.458; log-rank = 0) and *AQP4* (HR:1.191; log-rank = 0.003) expression (Figures 5B, D). In the GBM patient groups, there was no difference between the high and low expression groups of these selected genes (Figures 5A, C).

Our findings suggest that high expression of *AQP1* and *AQP4* may be a risk factor for poor prognosis in patients with LGG because they are significantly associated with reduced survival.

### 3.4 Functional enrichment analysis

Understanding the disease functioning from different levels of information is a crucial step in this line of work, as we look for potential regulators of AQPs only in gliomas.

The DAVID tool analysis generated two lists of dysregulated pathways from upregulated and downregulated genes in the glioma microenvironment. A list of genes with LogFC ≥ 1.0 revealed 64 altered pathways (Supplementary Table 1). Among them, 31 presented statistical significance (*p* < 0.05). Sixty-six pathways were identified in genes with negative LogFC values (Table 2). Among them, 30 were statistically significant.

**TABLE 2:** Correlation analysis between genes involved in the tyrosine metabolism pathway and AQP’s genes.

*AQP1*			*AQP4*	
Gene ID	Coeffici-ent	*P*-Value	Coeffici-ent	*P*-Value
**Gliomas (LGG and GBM)**				
*PNMT*	0.25	4.5e–17	0.31	4.5e–17
*ALDH1A3*	0.31	5.3e–17	0.17	5.3e–17
*TPO*	0.056	0.14	0.0062	0.87
*AOC2*	−0.21	3.7e–08	−0.15	3.7e–08
*GOT1*	−0.076	0.046	0.055	0.15
*HGD*	0.41	6.5e–29	0.14	6.5e–29
*ADH1B*	−0.063	0.1	0.079	0.039
*TYRP1*	0.12	0.0017	0.17	1.2e–05
*TH*	−0.01	0.79	−0.033	0.39

PathfindR analysis showed 113 pathways over-represented according to glioma differential expression (Supplementary Table 3).

Based on our previous study (Costa et al., 2019), where AQP4 was overexpressed both during central nervous system development and in human glioma cells and triiodothyronine (T3) played a negative role in this expression pattern, we chose tree-specific pathways in the downregulated list of the functional enrichment analysis: hsa00350 (Tyrosine metabolism [9 genes]); hsa04918 (Thyroid hormone synthesis [15 genes]); and hsa04919 (Thyroid hormone signaling pathway [7 genes]) (Supplementary Table 3). It is worth noting that choosing the genes from downregulated pathways was timely to antagonize the overexpression of AQPs as well as in our experimental previous study (*in vivo* and *in vitro*). In total, we identified 31 genes from the cellular pathways identified in the enrichment analysis.

### 3.5 Spearman’s correlation

We hypothesized that the expression of both AQP1 and AQP4 could be modulated by genes involved in thyroid function. Although still controversial, accumulated evidence suggests that thyroid function plays an important role in several pathological and non-pathological processes of the central nervous system, including gliomagenesis, cell migration and cerebral edema resolution (Schiera et al., 2021) having AQP4 as one of its action targets, as shown in two experimental circumstances, mentioned above, evidenced by our research group (Costa et al., 2019). Tyrosine, in turn, is the precursor amino acid of thyroid hormones by the iodination of tyrosine residues in thyroglobulin and has their levels altered in some disease status (Khaliq et al., 2015). Here, we attempted to identify potential regulators that could interfere with some degree of regulation of the disposition and function of AQP1 and AQP4 in tumor cells (Tables 2–4).

**TABLE 3:** Correlation analysis between genes involved in the thyroid hormones synthesis pathway and AQP’s genes.

*AQP1*			*AQP4*	
Gene ID	Coeffici-ent	*P*-Value	Coeffici-ent	*P*-Value
**Gliomas (LGG and GBM)**				
*PRKCG*	−0.075	0.049	0.17	4.8e–06
*ADCY1*	0.12	0.0023	0.18	1.2e–06
*ATP1B1*	0.23	1.2e–09	0.35	5.9e–21
*ADCY5*	−0.32	2.9e–17	0.0065	0.86
*TPO*	0.056	0.14	0.0062	0.87
*PLCB4*	−0.3	8.9e–16	−0.096	0.012
*TG*	0.18	4.3e–06	0.16	3.2e–05
*CREB3L3*	−0.082	0.033	0.1	0.0076
*IYD*	0.048	0.21	0.056	0.14
*GPX2*	−0.065	0.088	0.045	0.24
*PRKCB*	−0.18	1.3e–06	0.11	1.3e–06
*ADCY4*	0.068	0.076	−0.037	0.33
*ITPR1*	0.23	2.6e–09	0.26	2.6e–09
*ATP1A3*	−0.31	1.2e–16	−0.12	1.2e–16
*LRP2*	0.16	3.4e–05	0.22	3.4e–05

**TABLE 4:** Correlation analysis between genes involved in the thyroid hormones signaling pathway and AQP’s genes.

*AQP1*			*AQP4*	
Gene ID	Coeffici-ent	*P*-Value	Coeffici-ent	*P*-Value
**Gliomas (LGG and GBM)**				
*SIN3A*	−0.14	0.00041	0.14	0.00024
*HDAC1*	0.44	3.6e–34	0.15	7.6e–05
*MED24*	−0.24	2.1e–10	−0.22	5e–09
*ATP2A2*	−0.15	0.00013	0.16	3.5e–05
*MTOR*	0.27	3.3e–13	0.26	8e–12
*ACTG1*	0.096	0.012	0.029	0.45
*ACTB*	0.35	1e–20	0.038	0.32

Within the tyrosine metabolism pathway, five genes were correlated with AQP1 and AQP4, although only four met the statistical requirements already established: *PNMT*, *ALDH1A3*, *AOC2*, and *HGD* (Table 2).

In the thyroid hormone synthesis pathway, six genes were statistically significant (Table 3): *ATP1B1*, *ADCY5*, *PLCB4*, *ITPR1*, *ATP1A3*, and *LRP2*. Only four genes involved in the thyroid hormone signaling pathway (Table 4) were within the statistical inclusion criteria described in Subsection 2.5: *HDAC1*, *MED24*, *MTOR*, and *ACTB1*. All these genes are also listed and have potential actions on AQPs, as summarized in Table 5. Our data refer to the number of transcripts using Spearman’s correlation analysis (AQP1 and AQP4 vs. genes selected from the functional enrichment analysis). Thus, we can consider them regulators at the translational level.

**TABLE 5:** Genes involved in the thyroid hormone and tyrosine pathways.

Official symbol	Official full name
*PRKCG*	Protein kinase C gamma
*ADCY1*	Adenylate cyclase 1
*ATP1B1*	Atpase Na+/k+ transporting subunit beta 1
*ADCY5*	Adenylate cyclase 5
*TPO*	Thyroid peroxidase
*PLCB4*	Phospholipase C beta 4
*TG*	Thyroglobulin
*CREB3L3*	Camp responsive element binding protein 3 like 3
*IYD*	Iodotyrosine deiodinase
*GPX2*	Glutathione peroxidase 2
*PRKCB*	Protein kinase C beta type
*ADCY4*	Adenylate cyclase 4
*ITPR1*	Inositol 1,4,5—triphosphate receptor type 1
*ATP1A3*	Atpase Na+/k+ transporting subunit alfa 3
*LRP2*	LDL receptor related protein 2
*SIN3A*	Transcription regulator Family member A
*HDAC1*	Histone deacetylase 1
*MED24*	Mediator complex subunit 24
*ATP2A2*	Atpase Na+/k+ transporting subunit alfa 2
*MTOR*	Mechanistic Target of Rapamycin kinase
*ACTG1*	Actin gamma 1
*ACTB*	Actin beta
*PNMT*	Phenylethanolamine *N*-methyltransferase
*ALDH1A3*	Aldehyde dehydrogenase 1 family member A3
*AOC2*	Amine Oxidase Copper Containing 2
*HGD*	Homogentisate 1,2–dioxygenase
*ADH1B*	Alcohol dehydrogenase 1B
*TYRP1*	Tyrosinase-related protein 1
*TH*	Tyrosine Hydroxylase

All information was derived from the National Center for Biotechnology Information (NCBI) database. Available in: https://www.ncbi.nlm.nih.gov/guide/genes-expression/.

## 4 Discussion

In this study, we aimed to identify the expression pattern of *AQP1* and *AQP4* genes in human gliomas, as well as to highlight their regulatory potential within the cancer microenvironment.

We used RNA-seq as a search strategy to identify the differential expression of AQP1 and AQP4 transcripts in glioma tissues data compared to normal brain tissues data. Indeed, AQPs genes are overexpressed in patients with glioma. Among the glioma subtypes, *AQP1* and *AQP4* are overexpressed in astrocytoma (LGG) and classical glioma (GBM). OS analysis showed that both AQP genes can be used as prognostic factors for patients with LGG, confirming the results of previous studies and reinforcing their clinical value. We also observed a correlation between the expression of genes involved in the tyrosine and thyroid hormone pathways and AQPs.

Emerging evidence suggests a positive relationship between AQP1 and AQP4 expression and histological tumor grade and brain edema volume. Poor neurological prognosis has also been reported (Suzuki et al., 2018; Chow et al., 2020). Our results are supported by these findings, as aquaporin expression patterns increased when compared between molecular subtypes of gliomas: AQP1 is increased in other subtypes of LGG classification (astrocytoma); in contrast, AQP4 is increased in only the classical subtype GBM.

Some current studies have highlighted the regulatory function of AQPs on brain volume in many other neurological diseases besides tumors (Sun et al., 2022; Ohmura et al., 2023). In gliomas, both AQP1 and AQP4, when suppressed, offer a better prognosis for patients affected by these types of tumors by edema reduction (Faraj et al., 2022; Ohmura et al., 2023), facilitating the management of neurological deficits in the pre-and postoperative periods, with peritumoral edema being more relevant, in terms of concerns the aggressiveness of this cancer, rather than its extent, *per se* (Faraj et al., 2022). AQP1, for example, is upregulated in the peritumoral area, especially in the vessels surrounding reactive astrocytes in high-grade gliomas. In LGG, it showed an intense pattern of intratumoral expression (Zoccarato et al., 2021). The discovery of the glymphatic system was also important for the consolidation of AQPs as targets therapeutic in brain edema regulation (Ding et al., 2023).

From these findings it is possible to suggest that *AQPs* are perhaps more relevant in the tumor progression process, that is, the transformation of LGG into GBM through the formation of expansive edema, facilitating the invasion of tumor cells into healthy tissue. The determining factors in the survival rate of patients with GBM, in turn, are other and more diversified, in addition to the expression pattern of *AQPs* (Deb et al., 2012; Saito et al., 2022), which would explain our results from the survival analysis, where *AQP* genes only functioned as a risk factor for survival in low-grade gliomas.

AQP4 isoforms, known as M1 and M23, have been shown to change the aggregation/disaggregation state into orthogonal arrangements of particles (OAPs) and glioma cell survival. One study revealed that the isoform M23 reduced the invasion and proliferation of glioma cells to promote their apoptosis (Simone et al., 2019).

Some endogenous factors such as hormonal and metabolic changes also modulate AQP4 expression in human gliomas (Lan et al., 2017). Likewise, an increase in *AQP1* expression was observed during central necrosis and hypoxia, which are characteristics of GBM tumors (Hayashi et al., 2007; Honasoge and Sontheimer, 2013).

Gene expression is orchestrated by several endogenous and exogenous factors, such as DNA-binding transcription factors, regulatory proteins, and profile hormones (Bhat et al., 2021).

A study conducted by Nauman et al. (2004) evaluated the cellular concentrations of thyroxine (T4) and triiodothyronine (T3) in non-thyroidal illness syndrome (NTIS) patients with gliomas, where the thyroid stimulating hormone (TSH) serum concentration was within the normal range and T4/T3 levels were lower than normal (Nauman et al., 2004). They demonstrated that thyroid hormone levels were significantly lower in glioma tissues than in healthy tissues. In addition, both iodothyronine deiodinases (types 2 and 3) were higher in tumor tissues than in non-glioma tissues.

Some studies have shown that hypothyroidism is an important factor in the prognosis and progression of metastatic cancer in patients with brain cancer. For example, a cohort study (Berghoff et al., 2020) identified a beneficial role of this clinical condition in overall survival in patients with brain metastases, almost doubling life expectancy from the diagnosis of primary and metastatic cancer. Furthermore, the results of a clinical survey showed that almost 40% of patients with brain tumors evaluated had overt or subclinical hypothyroidism and 31% required hormone replacement (Faghih-Jouybari et al., 2018).

In pediatric patients, an important probability of developing thyroid dysfunction was found post-treatment with surgery, chemotherapy, and radiotherapy (Jin et al., 2018; Cosnarovici et al., 2020); moreover, abnormalities in thyroid function were found in patients with AQP4 antibody–seropositive optic neuritis (Zhao et al., 2017).

We previously identified a negative modulator of AQP4 protein and demonstrated that triiodothyronine (T3) treatment of GBM-95-line cells resulted in a slight reduction in cell migration (Costa et al., 2019); other studies have also evaluated the modulatory action of this hormone on the expression of AQP4 in stroke animal models (Sadana et al., 2015).

**FIGURE 6:**
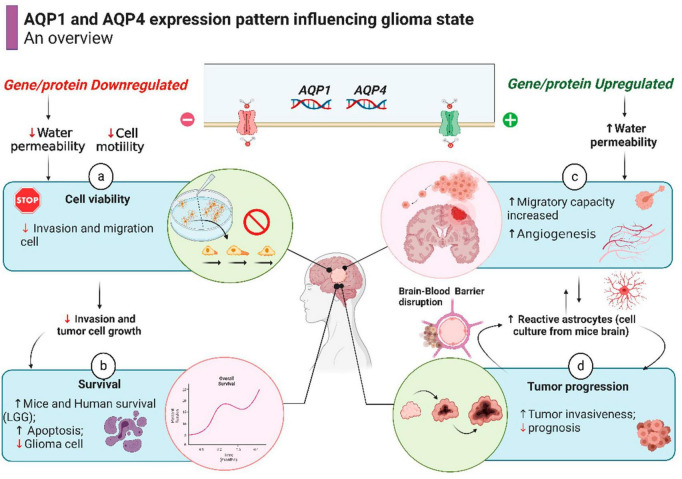
Schematic representation of cell processes involving AQPs activity in glioma state (already demonstrated by prior studies), resulting in better or worse outcome for a patient. The overall survival in human patient with LGG is shown in Figure 5. **Source: Subscription:** Individual, **Agreement number:** XZ26G6XU1D, **Journal name:** Frontiers in Neuroscience.

In recent years, a specific AQP4 inhibitor has been tested in rodents and human brain glioma to directly measure water efflux rate during tumorigeneses (Harrison et al., 2020; Rosu et al., 2020). A promising study conducted by Jia et al. (2023) found a correlation with water efflux in glioma cell cultures, rat and human glioma and cell proliferation as measured by magnetic resonance imaging. This discovery is in agreement with our results showing overexpression of AQP4 genes in gliomas, placing aquaporin 4 as an important biomarker to be used both in bioinformatics analyzes and in brain imaging as a potential therapeutic treatment of gliomas. A summary of what is hypothesized/proven about the modulation of the expression of AQPs 1 and 4 in gliomas is briefly outlined in Figure 6.

Therefore, according to our results, we suggest two new hypotheses: (1) As all patient data are post-mortem (including data other than this one), it is possible that standard cancer treatment led to hypothyroidism and subsequently downregulated the thyroid hormone pathways. This could result in the overexpression of AQP1 and AQP4 in the more aggressive glioma subtypes [astrocytoma (LGG) and classical, (GBM)], as we present in the differential expression results, (2) hypothyroidism would already be a clinical risk condition for the development of glioma; patients diagnosed early with the tumor would present a sub-clinical pattern of thyroid gland dysfunction. This condition would be accentuated by the anticancer therapeutic approach, and these patients would have the same outcome and genetic, and molecular profiles as mentioned in hypothesis 1.

Additional studies to clarify the specific inhibitors or promoters of these pathways, as well as transcription factors and post-transcription/translation modification methods, will help to identify the most appropriate therapeutic targets for AQP1 and AQP4 modulation, relationships between genes, and signaling pathways in gliomas.

To the best of our knowledge, this is the first study to suggest a modulatory interaction between the gene encoding AQP1 and pathways related to thyroid hormones.

## 5 Limitations

The brain tissue is substantially plastic and heterogeneous. Factors such as age and sex can influence their genetic constitution. Glial tumor characteristics may also change depending on the topographic region of the brain where the tumor occurs. The non-separation of categorical patient groups based on the aforementioned factors is a limitation of this study. Single-cell analysis from different glioma patient groups would help us to confirm the oncogenic role of the AQPs studied here, as well as their modulation by thyroid hormones.

## 6 Conclusion

To the best of our knowledge this study is the first to show a strong potential functional relationship between AQP1 and thyroid hormone pathways in brain tumors. The expression of AQP1 and AQP4 is significantly associated with a worse prognosis in patients with LGG. The molecular pathways and AQP1 and AQP4 genes identified here may be useful for the molecular diagnosis of gliomas and for screening new anti-tumor drugs for these malignant tumors.

## Data availability statement

The datasets presented in this study can be found in online repositories. The names of the repository/repositories and accession number(s) can be found in the article/Supplementary material.

## Author contributions

CM: Conceptualization, Formal Analysis, Investigation, Writing – original draft, Writing – review and editing. LR: Writing – review and editing. CC: Data curation, Writing – original draft. AX: Conceptualization, Data curation, Project administration, Supervision, Writing – original draft, Writing – review and editing.

## Footnotes

1
http://gepia2.cancer-pku.cn

2
http://timer.cistrome.org/

3
https://www.proteinatlas.org/

4
https://david.ncifcrf.gov/

5
https://portal.gdc.cancer.gov/
