# Supplementary figures and images for: Homeostatic status of thyroid hormones and brain water movement as determinant factors in biology of cerebral gliomas: a pilot study using a bioinformatics approach
Source: Front Neurosci. 2024 Feb 27;18:1349421. doi: 10.3389/fnins.2024.1349421 (PMC10927765; doi:10.3389/fnins.2024.1349421)

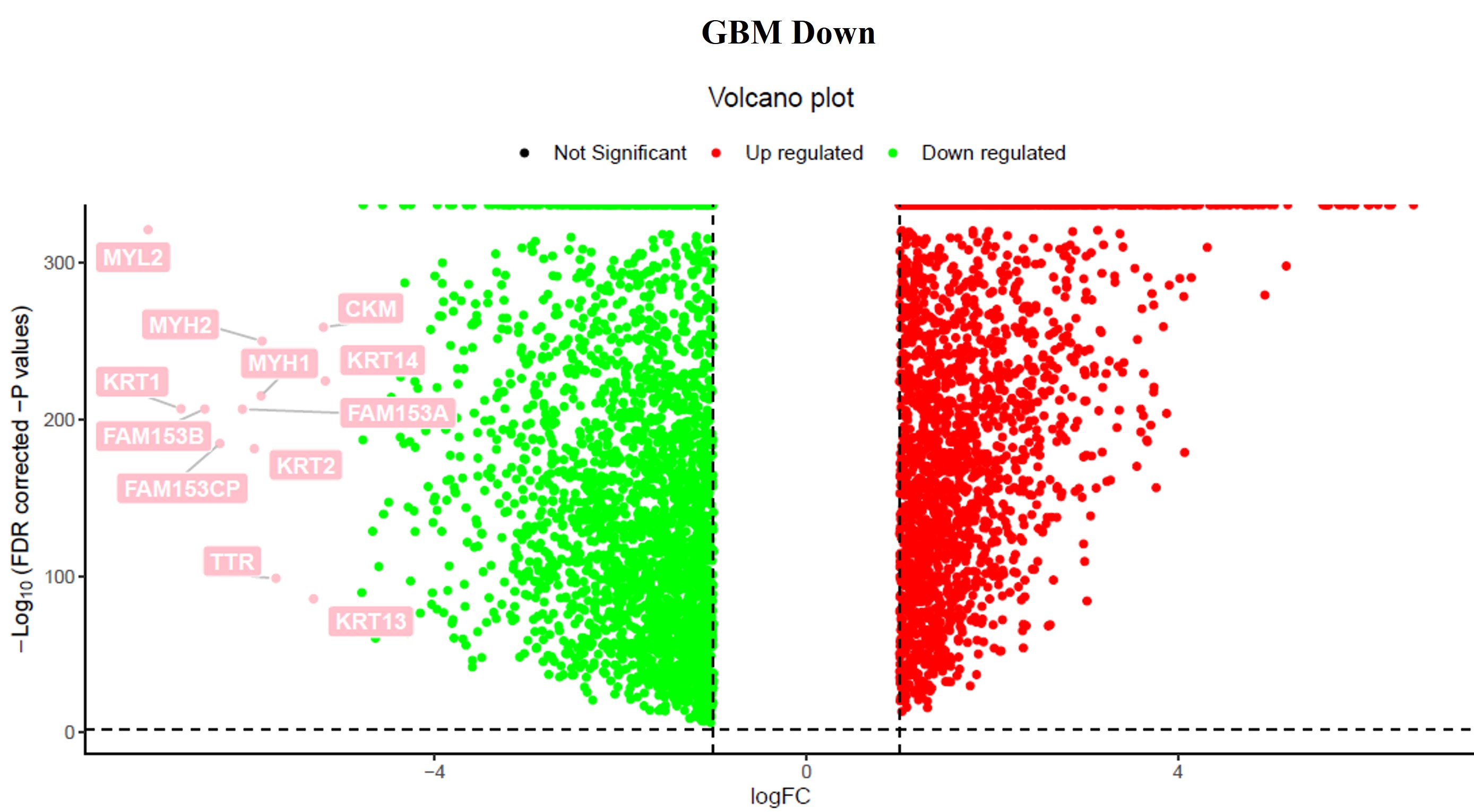

Supplement: Supplementary file 2 [file Image_1.JPEG]

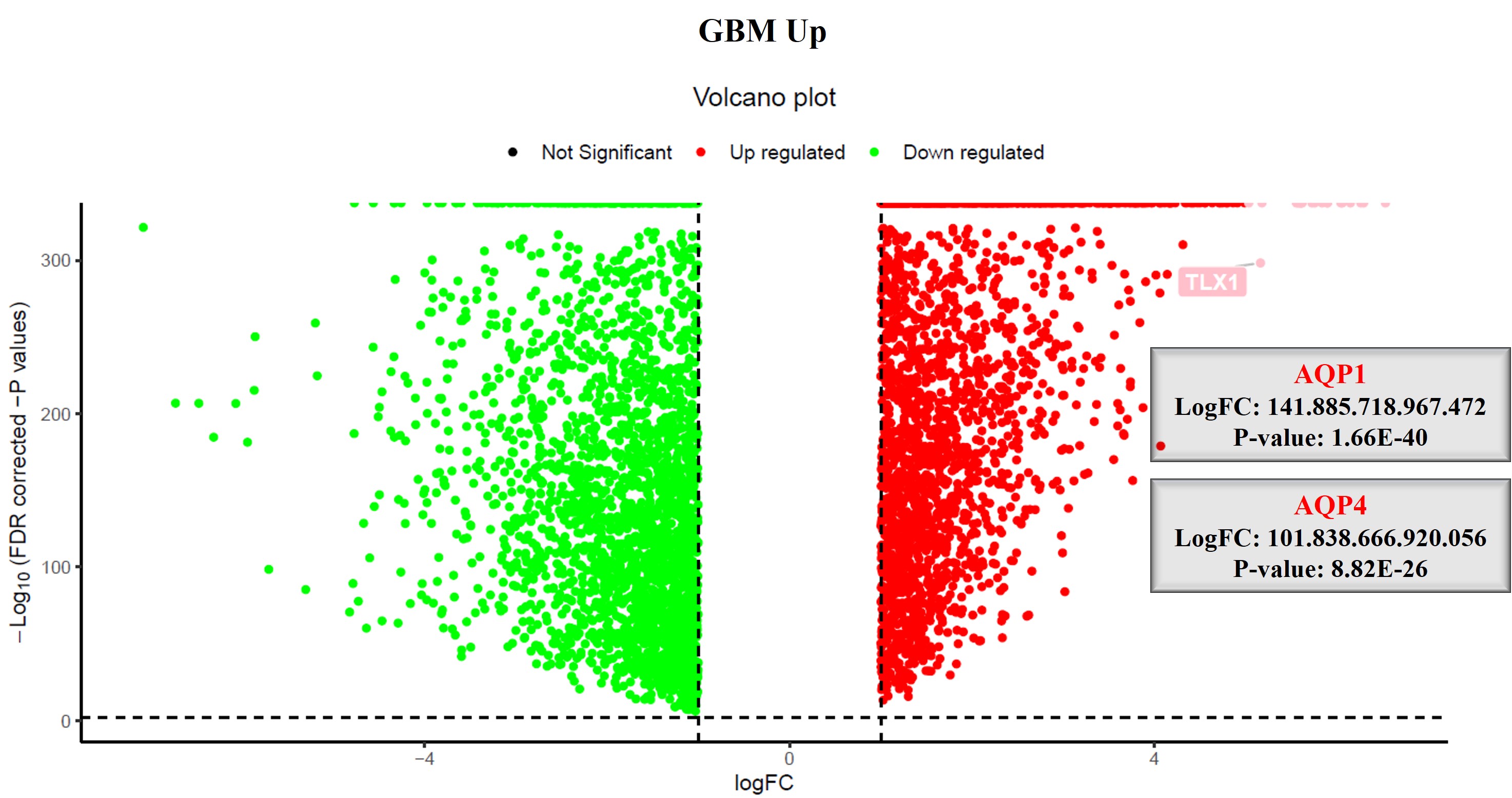

Supplement: Supplementary file 3 [file Image_2.JPEG]
